# Evaluating a Machine Learning Tool for the Classification of Pathological Uptake in Whole-Body PSMA-PET-CT Scans

**DOI:** 10.3390/tomography7030027

**Published:** 2021-07-29

**Authors:** Annette Erle, Sobhan Moazemi, Susanne Lütje, Markus Essler, Thomas Schultz, Ralph A. Bundschuh

**Affiliations:** 1 Department of Nuclear Medicine, University Hospital Bonn, 53127 Bonn, Germany; annette.erle@t-online.de (A.E.); susanne.luetje@ukbonn.de (S.L.); markus.essler@ukbonn.de (M.E.); ralph.bundschuh@ukbonn.de (R.A.B.); 2 Department of Computer Science, University of Bonn, 53115 Bonn, Germany; schultz@cs.uni-bonn.de; 3 Bonn-Aachen International Center for Information Technology (B-IT), 53115 Bonn, Germany

**Keywords:** prostate cancer (PC), prostate specific membrane antigen (PSMA), positron emission tomography (PET), computed tomography (CT), radiomics features (RFs), machine learning (ML)

## Abstract

The importance of machine learning (ML) in the clinical environment increases constantly. Differentiation of pathological from physiological tracer-uptake in positron emission tomography/computed tomography (PET/CT) images is considered time-consuming and attention intensive, hence crucial for diagnosis and treatment planning. This study aimed at comparing and validating supervised ML algorithms to classify pathological uptake in prostate cancer (PC) patients based on prostate-specific membrane antigen (PSMA)-PET/CT. Retrospective analysis of ^68^Ga-PSMA-PET/CTs of 72 PC patients resulted in a total of 77 radiomics features from 2452 manually delineated hotspots for training and labeled pathological (1629) or physiological (823) as ground truth (GT). As the held-out test dataset, 331 hotspots (path.:128, phys.: 203) were delineated in 15 other patients. Three ML classifiers were trained and ranked to assess classification performance. As a result, a high overall average performance (area under the curve (AUC) of 0.98) was achieved, especially to detect pathological uptake (0.97 mean sensitivity). However, there is still room for improvement to detect physiological uptake (0.82 mean specificity), especially for glands. The ML algorithm applied to manually delineated lesions predicts hotspot labels with high accuracy on unseen data and may be an important tool to assist in clinical diagnosis.

## 1. Introduction

Prostate Cancer is one of the leading malignancies in the whole world, with 1.3 million new cases in 2018. It is the second most common cancer in men worldwide [1] and is the fifth leading cause of cancer death [2]. The number of deaths is estimated to rise by 105.6% by 2040 [2]. While the overall 5-year-survival-rate reaches 98.0%, it decreases to 30.5% once metastases occur [3].

In order to diagnose the disease, detect possible progression and monitor therapy response, body scans are indispensable. Hybrid positron emission tomography/computed tomography (PET/CT) scans depict anatomical data on the one hand and functional information of tissues on the other hand. As a favorable biomarker for PET in prostate cancer patients, radiolabeled ligands to prostate-specific membrane antigen (PSMA) are used, being considered in many studies [4]. PSMA is expressed in prostate cells in very low concentration and overexpressed in an increasing degree on prostate cancer cells [5]. Therefore, it allows distinguishing between benign and malignant tissues. For PET diagnosis, small molecules targeting PSMA and labeled with the positron emitters, gallium-68 (^68^Ga) [6] or fluorine-18 (^18^F) [7], are utilized. The combination of PSMA-PET and CT allows allocating metastasis accurately [8,9].

So far, analysis of PSMA-PET/CT image data, performed by specialists in nuclear medicine, is mainly done manually based on experience. This has several disadvantages, being time-consuming and error-prone with high numbers of inter- and intra-observer variability [10]. Here, the use of computer-aided diagnosis (CAD) has several advantages: it can improve diagnostic precision, accelerates analysis, facilitates the clinical workflow, lowers human resource costs, and even may predict prognosis, and last but not least, if trained with proper datasets, can compensate for inter- and intra-observer variability [11]. Machine learning (ML) algorithms base their analysis on very large amounts of unstructured information (“big data” [12]), which allows the recognition of complex patterns. Especially in imaging, their application has shown to be very successful [13,14,15]. In hybrid imaging, the implementation of artificial intelligence (AI) is a promising field with application in a wide range of clinical sectors, e.g., Morbus Alzheimer in neurology, lung cancer, multiple myeloma, and prostate cancer in oncology [10,11]. Analysis of textural parameters in standard clinical image data allows performing segmentation and characterization of tissues as well as being useful in the fields of prediction and prognosis, leading the way to individually tailored therapies in the future [16,17,18]. In the field of prostate cancer, ML methods are furthermore already used and growing, e.g., in treatment, histopathology and genetics [13,14]. Khurshid et al. have shown that the two textural heterogeneity parameters, entropy and homogeneity, correlate with pre/post-therapy prostate-specific antigen (PSA) levels. A higher level of heterogeneity seems to predict a better response to PSMA therapy and may in the future allow a pre-therapeutic selection of responders to the treatment [19]. Moazemi et al. have consecutively shown that these parameters also have prognostic potential for the overall survival of prostate cancer patients [20]. In summary, the software could take over and improve the whole process in the future, enabling specialists to focus on more important tasks. For such CAD, including decision support algorithms, at first, the detection of pathological lesions is necessary, followed by an analysis of the radiomics features in these lesions. Although the automatic segmentation of high tracer uptake would be an essential step towards such a clinical decision support tool, in this study, we focus on the automated classification of pathological vs physiological uptake using radiomics features from manually segmented hotspots and leave the automated segmentation as future work. When present, such an automated system would enhance the procedure of the management of PC patients in terms of time and effort.

Therefore, in the present work, we further compare and evaluate supervised ML-based algorithms for classifying hotspots in PSMA PET/CT, which have shown their potential before [21]. Additionally, to verify the significance of the existing algorithm, the training cohort was gradually extended to find out with how many subjects our model would generalize. The aim of this study was to quantify the accuracy of the algorithm as applied to unseen sets of data, especially focusing on enhancing the true classification of hotspots with physiological uptake. To this end, first, the patients’ scans are manually annotated to provide data. Then, python software undertakes the task of classifying uptakes into two categories (pathological vs physiological). Finally, the output is verified and reviewed with an independent test cohort.

## 2. Materials and Methods

### 2.1. Patients and Volume of Interest (VoI) Delineation

Data of 87 patients with histologically proven prostate cancer were included in this analysis. All patients received a PSMA-PET/CT examination due to clinical reasons, either for staging or treatment control. PET/CT examinations were performed 40 to 80 min after the intravenous injection from 98 to 159 MBq in-house produced ^68^Ga-HBED-CC PSMA using a Siemens Biograph 2 PET/CT machine (Siemens Healthineers, Erlangen, Germany). First, a low-dose CT (16 mAS, 130 kV) was performed from the skull to mid-thigh, followed by the PET imaging over the same area with 3 or 4 min per bed position depending on the weight of the patient. PET data were reconstructed in 128 × 128 matrices with 5 mm slice thickness, while CT data were reconstructed in 512 × 512 matrices with 5 mm slice thickness. For PET image reconstruction, the attenuation-weighted ordered subsets expectation-maximization algorithm implemented by the manufacturer was used, including attenuation and scatter correction based on the CT data. Additionally, applied by the manufacturer, a 5 mm post-reconstruction Gaussian filtering was used for smoothing of all the input images prior to the ML analyses. All patients gave written and informed consent to the diagnostic procedure. Due to the retrospective character of the data analysis, an ethical statement was waived by the institutional ethical review board according to the professional regulations of the medical board of Nordrhein-Westfalen, Germany.

**Figure 1:**
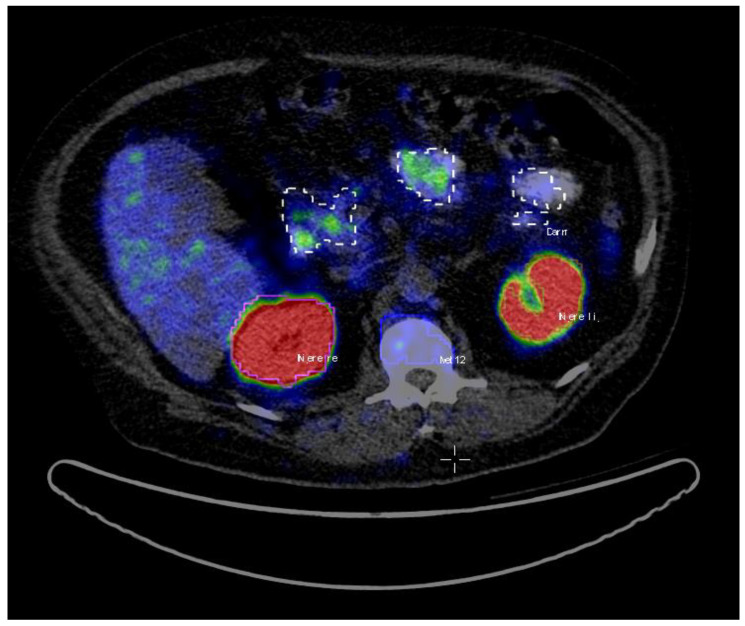
A sample screenshot from the hotspots’ delineation step using InterView Fusion Software (Mediso, Budapest, Hungary) [22]. The hotspots are identified and delineated slice by slice as 3D volumes of interests (VoIs) by an experienced nuclear medicine physician (the hotspot names translation from German: Niere = kidney, Darm = gut. Moreover, Met stands for metastasis).

A total of 72 patients were assigned as the training dataset. The patients’ average age was 71 (range: 48–87), and the average Gleason score was 8 (range: 6–10) (Table 1). The PET/CT images data were analyzed using Interview Fusion Software by Mediso Medical Imaging (Budapest, Hungary) [22]. All the hotspots have been identified based on fused PET and CT data. Volumes of interest (VoIs) were manually delineated with a brush tool in the PET images slice by slice (Figure 1). The criteria to choose an uptake was the visible tracer uptake without any predefined threshold. In a second step, the hotspots were classified as pathological or physiological, corresponding to the location they were situated in. The hotspots included primary prostate cancer and metastases in the skeletal system, lymph nodes, as well as physiological uptake in kidneys, liver, glands, gastrointestinal tract (gut) etc. A total number of 2452 hotspots were marked and then categorized as either pathological (total of 1629) or physiological (total of 823).

In total, 15 remaining patients with similar ranges of age, Gleason score, and PSA level as the patients in the training cohort were assigned to the test group for testing the ML algorithm (Table 1). First, the scans were again analyzed in the same way with Interview Fusion software as described above. For every patient, 5 to 10 pathological hotspots were delineated and tracer uptake in all glands; 5 physiological hotspots and uptake in the liver. Beforehand, glands proved to be difficult for the algorithm to classify whether physiological or pathological. This analysis resulted in 331 hotspots with 128 pathological and 203 physiological lesions.

For each hotspot, a total of 77 radiomics features were calculated using InterView FUSION software (Mediso Medical Imaging, Budapest, Hungary). The features include first/higher order statistics, textural heterogeneity parameters, and zone/run length statistics features. The complete list of the features is provided in Table 2.

### 2.2. Training and Classification

For the ML analyses, the question of pathological versus physiological uptake was mapped to the so-called supervised ML problem [23]. This sort of ML algorithm is applied when part of the study cohort already includes complete information on input variables (in our case, PET/CT hotspots and their corresponding radiomics features) as well as ground truth labels (in our case, pathological vs physiological). Thus, for training and classification purposes, an in-house developed software in Python V.3.5 was used. Initially, we used a 30-subjects subset of the training dataset of 72 subjects to pre-set, tune, and compare our machine learning classifiers from SciKitLearn library [24] (linear kernel support vector machine (SVM), as well as ExtraTrees [25] and random forest as classifiers based on decision trees [26]), which already showed their significance [21].

In the first round of training, cross-validation (CV) is applied with KFold with 3 folds to tune hyperparameters of the classifiers and identify the best performing one. To this end, at each CV step, the C and Gamma parameters of the linear SVM as well as the min_sample_leaf and max_depth of the decision tree-based algorithms are tuned using grid search. For the hyperparameter tuning, standard ranges of the hyperparameters are applied. For example, for C, the range from 2^−5^ to 2^13^ is used, and for max_depth, the range from 1 to 10 is used for the grid search. This has resulted in the best combination of the hyperparameters for each classifier and helped to identify the best classifier based on the performance metrics AUC, sensitivity (SE), and specificity (SP). We further quantified the performances of the classifiers on the test cohort to end up with the best algorithm, which was Extra Trees. From this point, we used Extra Trees with its tuned hyperparameters (n_estimators = 250, max_depth = 20, min_samples_leaf = 1) to investigate how the algorithm would generalize as the size of the training cohort increases.

For the next step to assess the generalizability of the algorithms, we started with the first initial cohort of 30 patients for training the algorithm. Then, we added the data from the second training cohort (42 patients), one patient at a time and with a randomized order (thus, the sizes of the training subsets varied from 30 to 72). It means, in each training step, a random subset of the combination of the two training cohorts was chosen, and each time the size of the subset was increased by one patient. Furthermore, we repeated the classification task 100 times with a bootstrapping approach to calculate the accuracy measures at each training step. As we aimed at assessing the performance of our algorithm on unseen data, in each step, we calculated the prediction accuracies (AUC, sensitivity (SE), and specificity (SP)) on the validation set (the hold-out set with 15 subjects). Finally, we report the mean and standard deviation (std) of the accuracy metrics to give an overview of how increasing the size of the training cohort affects the classification performance metrics when trained by the training cohorts of 30 to 72 subjects and tested by the test cohort of 15 subjects. The performance metrics were calculated at each training step based on the prediction scores of the ML classifier as trained by the training cohort and tested by the test cohort, then averaged along 100 bootstraps.

To minimize the risk of overfitting, first, the dataset including feature vectors of training and test subjects were normalized, using the MinMax standardization method. This method maps the input variables into the range between 0 and 1 to compensate for inconsistent variable ranges. As a result, variables with very large or very small values would not affect the classifier performance. For this study, we chose to apply both cross-validations to identify the best classifier and bootstrapping with replacement and resampling on the training set to better estimate the population statistics.

## 3. Results

### 3.1. Pathological and Physiological Hotspots

In the 72 patients’ scans of the training cohort, 2452 hotspots have been identified and marked. Out of these, 823 hotspots have been classified as physiological versus 1634 as pathological. In the 15 patients of the control cohort, 331 areas with increased uptake have been defined. Table 3 shows the detailed distribution of hotspots in the training cohort, and Table 4 shows the summarized distribution of the hotspots in the train and test cohorts. In the training cohort of 72 patients, the category “others” denotes 33 lesions in other organs such as the spleen and lung.

### 3.2. Patient Validation

**Figure 2:**
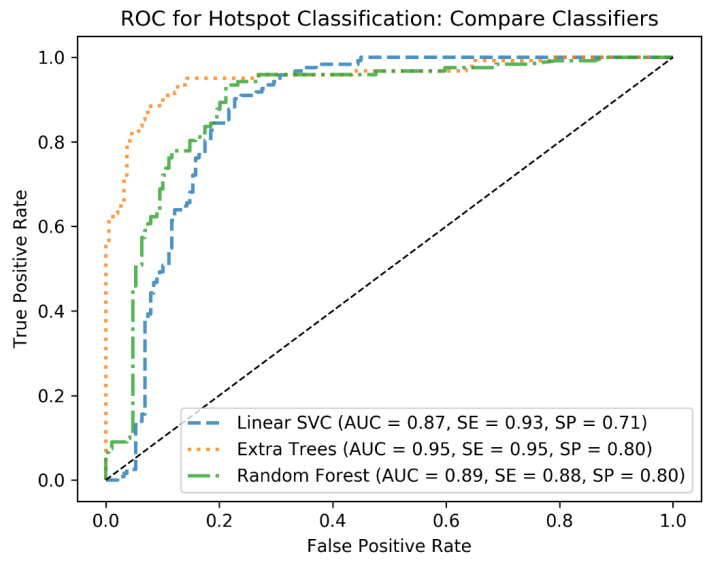
The receiver operating characteristic (ROC) curves to compare three classifiers. The classifiers are ranked after tuning in the cross-validation step and trained with the first training cohort with 30 subjects and then applied to the test cohort.

As a result of the cross-validation step, the Extra Trees classifier outperformed other classifiers (see Figure 2). The Extra Trees classifier trained with the data of 30 patients resulted in an AUC of 0.95, a sensitivity of 0.95, and a specificity of 0.80. As expected, extending the training cohort by incrementing the sample size, one patient after another, resulted in improved accuracy measures until it reached its overall maximum as the data from the 72nd subject was imported (0.98 AUC, 0.97 sensitivity, and 0.83 specificity). Figure 2 gives an overview of how different classifiers performed on the first training cohort, and Table 5 shows the mean and standard deviation of the accuracy measures along all the training steps. Based on the training data of 72 subjects and the validation cohort of 15 subjects, 125 of the 128 lesions defined by the reader as pathological were identified as pathological as well, which means a sensitivity of 0.97. Additionally, the physiological uptake was classified with high precision. While the algorithm showed accurate predictions of liver, kidneys, GUT, etc., specificity regarding salivary glands showed to be more difficult with a figure of 0.82. Especially in sublingual and lacrimal glands, there was a high rate of false positives (9/19 and 7/19). Other glands were easier to identify as physiological, resulting in a total of only 2 (submandibular gland) and 1 (parotid gland) false positives. The complete overview is presented in Table 6. As pathological prostate uptake was only present in 14 subjects from the training cohort, we could not analyze the prediction performance on the test cohort for this category. However, analyzing the 14 prostate hotspots from the training cohort, we achieved a sensitivity of 0.92 (13/14 true positives). 

## 4. Discussion

The application of ML-based image analysis would implicate several benefits for patients. First of all, this examination is fast and non-invasive. Second of all, it could be used for therapy prediction and differentiation between low and high-risk patients [27] and for prediction of overall survival [20], as already published before. It could furthermore be the base of individual therapy and treatment tailoring in the future, combining the technique with analysis of textural parameters. Furthermore, the presented ML-based approach and the outcoming results confirm the potential of the ML methods for the analysis of newly day-to-day arriving patients’ data.

The problem with the smaller set of data was that more physiological hotspots were recognized as pathological, i.e., a high rate of false positives. Results with the bigger set of data have shown to be more specific though. However, the obtained prediction accuracies after increasing the training cohort size showed the robustness of the algorithms. In addition, it is important to note that we have a very high number of hotspots per patient (34 hotspots per patient on average). Therefore, even with a low number of patients we have, significant numbers of lesions.

As a matter of fact, overfitting is an important concern in ML studies, especially when the number of features exceeds the number of samples. In this study, we included 2452 hotspots data (n_samples = 2452) and 77 radiomics features (n_features = 77). We also applied MinMax standardization. We had already applied cross-validation in the previous study [21] to identify the best performing ML classifier and tune its hyperparameters. In this study, we applied Bootstrapping to better estimate the population statistics. To conclude, all these precautions had been made to avoid overfitting.

In this analysis, we could also identify in which locations the most problems appear in the AI algorithm. In fact, the categorization of glands proved to be difficult with a specificity of just 0.82. 19 out of 111 glands were identified as metastasis, especially sublingual (9/19) and lacrimal (7/19) glands. The reason why the ML algorithm performs poorer on glands might be caused by the fact that, from the data-driven point of view, the radiomics features of the glands seem to be in a similar range as for the pathological uptake. Therefore, this must be a topic of further improvement of the algorithm. However, this limitation of the algorithm seems to be acceptable for this feasibility study as the head is only in very far spread tumor disease, a typical location of metastases. For other organ uptakes as kidney and bladder, a specificity of 1.0 was reached. Even uptake in the GUT approached a result just as high with a specificity of 0.97. The performance of the ML classifier to classify pathological prostate uptake was also quantified as high (0.92 sensitivity). However, as no pathological uptake was annotated in the test cohort, we could not analyze it on the test step.

As the goal of the presented study was to show the feasibility and power of the lesion classification in PSMA-PET data, so far, we used manual segmentation. For fast data processing in clinical routine in future segmentation needs to be performed in an automatic or at least semi-automatic manner, e.g., as described for bone lesions in [28]. However, this topic was beyond the scope of this study, and as no satisfying algorithms for total lesion segmentation in PSMA-PET are available, we decided for a manual segmentation not to alter our results by problems with a not fully validated segmentation process. Even as manual segmentation shows several problems, e.g., interobserver variability, it is widely accepted in this context [19,29].

As one of the limitations of the current study, the gold standard, which is in our case, the human-experienced nuclear medicine physician, should be discussed. For the detection of pathological uptake, naturally, histopathology findings should be preferred. However, we had nearly 2500 hotspots in our training cohort of 72 patients, meaning nearly 35 hotspots per patient, including physiological uptake. Therefore, there is no option and justification for performing such a high number of biopsies. This is a general limitation of this kind of study.

Another general limitation that needs to be taken into account is that the whole study was performed on data of the same scanner. Although this was not part of the current study, in further steps, it should be analyzed how specific training and results are according to changes in scan devices or scan protocols. Such questions should be dealt with best in multicentric studies.

For further studies in ML analysis of medical image data, prostate cancer seems to be an ideal subject having a high and increasing prevalence. It should be easy to acquire “big data” and so to train, develop, and improve the algorithm. In the end, a large group of people would benefit from this study.

## 5. Conclusions

Computer-aided decision support systems analyzing radiomics features from pre-therapeutic 68Ga-PSMA-PET-CT scans and leveraging state-of-the-art supervised machine learning methods have shown their significance to identify pathological uptake in patients with advanced prostate carcinoma even on unseen data. Further improvement, however, should be done in the identification of small glands and in cross-validation of different PET scanners and perhaps other centers to overcome limitations of interobserver variability and problems due to scan specifications.

## Figures and Tables

**Table 1:** The age, Gleason score, and PSA level ranges for the patients in the training and test cohorts.

Parameter	Training Group			Test Cohort		
Name	Average	Min	Max	Average	Min	Max
Age	71	48	87	77	63	87
Gleason Score	8	6	10	8	6	10
PSA (ng/mL)	438.72	4.73	5910.0	660.62	1.2	5400.0

**Table 2:** A list of the radiomics features from both positron emission tomography (PET) and computed tomography (CT) modalities. Please note that the metabolic tumor volume (MTV) is PET-specific. This table is already published in [20].

First or Higher Order Statistics	Shape and Size	Textural	Volumetric Zone Length Statistics	Volumetric Run Length Statistics
Deviation	Max. Diameter	Entropy	Short Zone Emphasis	Short Run Emphasis
Mean		Homogeneity	Long Zone Emphasis	Long Run Emphasis
Max		Correlation	Low Grey-Level Zone Emphasis	Low Grey-Level Run Emphasis
Min		Contrast	High Grey-Level Zone Emphasis	High Grey-Level Run Emphasis
Sum		Size Variation	Short Zone Low Grey-Level Emphasis	Short Run Low Grey-Level Emphasis
PET-MTV		Intensity Variation	Short Zone High Grey-Level Emphasis	Short Run High Grey-Level Emphasis
Kurtosis		Coarseness	Long Zone Low Grey-Level Emphasis	Long Run Low Grey-Level Emphasis
		Busyness	Long Zone High Grey-Level Emphasis	Long Run High Grey-Level Emphasis
		Complexity	Zone Percentage	Grey-Level Non-Uniformity Run Length Non-Uniformity
				Run Percentage

**Table 3:** The distribution of hotspots throughout the training cohort subjects.

Patient ID	Metastasis	Bladder	Gut	Kidney	Ureter	Parotid Gland	Lacrimal Gland	Sub-Mandibular Gland	Sub-Lingual Gland	Prostate	Liver	Other
1	11	1	1	2	2	2	2	0	0	1	1	2
2	26	1	1	2	0	0	1	2	2	1	0	0
3	21	0	1	2	0	0	2	2	0	0	1	0
4	25	0	0	2	2	2	0	0	1	0	0	0
5	15	1	2	2	0	2	0	2	1	0	1	4
6	28	0	1	2	2	1	2	2	0	1	1	0
7	30	1	1	2	2	0	2	0	0	0	1	0
8	20	1	1	2	0	2	1	2	1	1	1	0
9	16	0	1	2	2	2	1	0	1	0	1	3
10	25	0	1	2	1	0	1	0	0	0	1	2
11	11	1	2	2	2	2	2	2	1	1	0	0
12	19	1	0	2	1	1	0	1	0	1	1	1
13	25	1	1	2	0	2	2	1	0	1	1	1
14	34	0	1	2	2	2	0	0	1	0	1	0
15	11	1	1	2	0	0	0	2	1	0	1	0
16	35	1	1	2	2	2	0	1	0	1	1	0
17	18	1	2	2	1	2	2	2	0	0	1	0
18	17	1	1	2	2	0	1	0	1	1	1	6
19	7	0	1	2	2	2	0	0	2	1	0	0
20	27	1	1	2	2	0	2	2	0	1	1	5
21	14	1	1	2	0	0	0	2	1	0	0	0
22	22	1	1	1	1	2	2	0	2	1	1	0
23	18	0	0	2	1	0	2	0	0	0	1	0
24	19	0	2	2	2	2	2	0	0	0	1	1
25	31	0	1	2	2	1	1	2	2	1	1	0
26	12	1	1	2	0	2	2	0	1	0	1	1
27	29	0	0	2	2	0	2	0	0	0	1	0
28	43	0	0	2	0	2	0	0	1	0	0	0
29	13	1	2	2	1	2	0	0	2	0	1	0
30	29	1	1	2	2	1	2	0	0	1	1	0
31	13	1	1	2	2	2	2	2	0	0	1	0
32	24	1	1	2	0	2	2	2	2	0	0	0
33	24	1	1	2	0	2	2	2	2	0	0	0
34	22	1	0	2	0	2	2	2	2	0	0	0
35	13	1	2	2	0	2	2	2	1	0	1	0
36	30	1	1	2	0	2	2	2	0	0	0	0
37	30	1	1	2	2	2	2	2	0	0	0	0
38	15	1	1	2	0	2	2	2	1	0	0	0
39	21	1	1	2	2	2	2	2	1	0	1	0
40	25	1	1	2	0	2	2	0	0	0	1	0
41	10	1	2	2	0	2	2	2	2	0	0	0
42	20	1	0	2	0	2	2	2	0	0	0	0
43	23	1	1	2	0	2	2	2	0	0	1	0
44	36	1	1	2	0	2	2	2	1	0	1	0
45	11	1	1	2	0	2	2	2	1	0	0	2
46	34	1	1	2	2	2	2	2	0	0	0	0
47	19	1	1	2	0	2	2	2	0	0	1	0
48	15	1	1	2	2	2	2	2	2	0	0	0
49	7	1	1	2	2	2	2	2	2	0	0	1
50	28	1	1	2	2	2	2	2	0	0	1	0
51	10	1	1	2	0	2	2	2	2	0	0	1
52	26	1	1	1	0	2	2	2	2	0	0	0
53	14	1	1	2	0	2	2	2	0	0	1	0
54	23	1	1	2	2	2	2	2	2	0	0	2
55	31	1	1	2	3	2	2	2	2	0	0	0
56	12	1	1	2	0	2	2	2	2	0	0	0
57	27	1	1	2	0	2	2	2	2	0	0	0
58	45	1	1	2	0	2	2	2	1	0	1	0
59	10	1	1	2	0	2	2	2	2	0	0	0
60	26	1	1	2	0	4	2	2	2	0	0	0
61	20	1	1	2	1	2	2	2	2	0	1	1
62	29	1	1	2	0	2	1	2	0	0	0	0
63	29	1	1	2	0	2	2	2	2	0	0	0
64	26	1	3	2	0	2	2	2	2	0	1	0
65	20	1	1	2	0	2	2	2	2	0	1	0
66	19	0	1	2	0	2	2	2	1	0	1	0
67	47	1	1	2	1	2	2	2	0	0	0	0
68	16	1	2	2	0	2	0	2	1	0	0	0
69	25	1	2	0	0	2	2	2	2	0	0	0
70	21	0	1	2	0	2	2	2	2	0	0	0
71	42	1	1	2	0	2	2	2	0	0	1	0
72	31	1	1	2	0	2	2	2	2	0	0	0
total	1620	58	76	140	57	122	115	107	71	14	39	33

**Table 4:** The distribution of the hotspots over different body organs in the training and test cohorts. (* the others category refers to the rest of organs in which hotspots were located).

Hotspot Category	Training Cohort 72 Patients	Test Cohort 15 Patients
Metastases	1620	128
Bladder	58	13
Kidney	140	30
Salivary Gland	415	111
Prostate	14	-
gut	76	33
liver	39	15
ureter	57	1
Others *	33	-
Total of hotspots	2452	331

**Table 5:** The mean and standard deviation (std) values of the area under the curves (AUCs), sensitivities, and specificities achieved as the training cohort was extended.

Accuracy Metric	Mean	Std
AUC	0.98	0.002
Sensitivity	0.97	0.004
Specificity	0.82	0.02

**Table 6:** The results of the predictions on the test cohort. (GUT: gastrointestinal tract).

Category	1/Pathological	0/Physiological	Total	Specificity
bladder	0	13	13	1.00
glands	19	92	111	0.82
GUT	1	32	33	0.97
liver	0	15	15	1.00
kidney	0	30	30	1.00
ureter	0	1	1	1.00
metastases	125	3	128	0.97

## Data Availability

The data are not publicly available because, according to German “Datenschutz Grundverordnung 2016/679”, patient data including images or part of images can only be used/seen within the hospital and by personnel of these hospitals if the patient has not agreed specifically to publish it. As this is a retrospective analysis, we have no agreement of the patients that we can make the data available openly, just that the data can be evaluated for studies.

## Abbreviations

PC	prostate cancer
PSMA	prostate specific membrane antigen
PSA	prostate specific antigen
PET	positron emission tomography
CT	computed tomography
RFs	radiomics features
ML	machine learning
AI	artificial intelligence
AUC	area under the curve
SE	sensitivity
SP	specificity
VoI	volume of interest
gut	gastrointestinal tract
